# A multi-task deep learning framework for simultaneous prediction of microsatellite instability and tumor mutational burden in gastric cancer from histopathological images

**DOI:** 10.3389/fonc.2026.1755232

**Published:** 2026-06-08

**Authors:** Yazhou Chang, Haoyue Chang, Yaping Lv, Shuxue Xi, Jialiang Yang, Bingzhi Wang, Xiaohao Zheng, Yibin Xie

**Affiliations:** 1 Department of Pancreatic and Gastric Surgery, National Cancer Center/National Clinical Research Center for Cancer/Cancer Hospital, Chinese Academy of Medical Sciences and Peking Union Medical College, Beijing, China; 2 Shanxi Medical University School of Forensic Medicine, Forensic Medicine, Taiyuan, China; 3 Geneis Beijing Co, Ltd, Beijing, China; 4 Academician Workstation, Changsha Medical University, Changsha, China; 5 Department of Pathology, National Cancer Center/National Clinical Research Center for Cancer/Cancer Hospital, Chinese Academy of Medical Sciences and Peking Union Medical College, Beijing, China; 6 Department of General Surgery, Beijing Friendship Hospital, Capital Medical University, Beijing, China

**Keywords:** deep learning, gastric cancer, histopathological images, immunotherapy, microsatellite instability, tumor mutational burden

## Abstract

**Background:**

The clinical management of gastric cancer (GC) increasingly relies on the biomarkers microsatellite instability (MSI) and tumor mutation burden (TMB) to identify patients likely to benefit from immunotherapy. However, their ubiquitous adoption is hampered by the high cost and complexity of next-generation sequencing. We hypothesized that a single deep learning model could simultaneously and accurately predict both biomarkers directly from routine histopathology slides, offering a transformative, cost-effective diagnostic tool. We aim to develop a multi-task deep learning framework to simultaneously predict MSI and TMB using routine histopathological images and clinical data.

**Methods:**

We presented a novel, interpretable, multi-task deep learning framework that concurrently predicted MSI and TMB status. Our model innovatively integrated whole slide images (WSIs) and clinical data in an end-to-end architecture. It employed a pre-trained ResNet50 for feature extraction, an attention mechanism to identify predictive image regions, and a Multimodal Compact Bilinear Pooling (MCBP) layer to fuse these image features with structured clinical data (gender, age, T/N/M stage). The model was trained on 312 patients from The Cancer Genome Atlas (TCGA). Furthermore, to ensure robustness, an expanded independent external validation cohort of 121 GC patients from our local center was incorporated from the Cancer Hospital, Chinese Academy of Medical Sciences.

**Results:**

The multimodal framework achieved robust performance in cross-validation, achieving area under the curve (AUC) values of 0.828 for MSI and 0.836 for TMB on the internal TCGA test set, outperforming standard models like ResNet18 and VGG. While the model achieved high AUCs internally, performance on the external validation set showed a moderate decrease due to domain shifts, yielding an AUC of 0.78 for MSI and 0.74 for TMB. Model interpretability was achieved through attention heatmaps, which revealed a significant spatial concordance between regions predictive of MSI and TMB from Quantitative spatial analysis, providing novel biological insight and validating our multi-task design.

**Conclusion:**

This work establishes the feasibility and accuracy of a unified, multi-task deep learning framework for the concurrent prediction of key immunotherapy biomarkers in gastric cancer. By leveraging routinely available histopathological images and clinical data, our method represents a significant innovation with immediate potential to lower the barrier to precision oncology in clinical practice. Our framework provides a cost-effective, preliminary screening tool for MSI and TMB in GC. Although external validation highlights challenges in generalizability across different scanners, this approach shows promise in triaging patients for immunotherapy.

## 1 Introduction

Gastric cancer (GC) represents a major global health burden, ranking as the fifth most common malignancy and the third leading cause of cancer-related mortality worldwide (1, 2). The disease demonstrates significant geographic disparity, with Eastern Asia exhibiting the highest attributable mortality rates (3). Notably, the overall 5-year survival rate remains as low as 32% (4), a poor prognosis largely driven by the fact that over 50% of patients are diagnosed at advanced or metastatic stages. This clinical reality underscores the urgent need to develop more effective and accessible treatment strategies.

In this context, immune checkpoint inhibitors (ICIs) have emerged as a revolutionary therapeutic option for advanced or metastatic gastric cancer. A growing body of evidence confirms that ICIs can significantly prolong survival in a subset of patients with advanced GC, highlighting their potential to reshape the treatment landscape. For example, anti-PD-1/PD-L1 drugs have demonstrated improvements in the overall survival (OS) of GC patients and prolonged treatment response (5, 6). Nevertheless, recent randomized trials have indicated that not all GC patients exhibit sensitivity to ICIs (7, 8). Hence, the development of predictive biomarkers for ICI response is urgently needed to enhance the therapeutic efficacy of ICIs in advanced GC. Nonetheless, a definitive biomarker for predicting the efficacy of ICIs is still lacking.

Presently, the three primary biomarkers for immunotherapy are PD-L1, microsatellite instability (MSI), and tumor mutation burden (TMB), with MSI and TMB-high (TMB-H) appearing to be correlated (9). MSI and TMB are critical for predicting the efficacy of immunotherapy. MSI is a hyper-mutative phenotypic condition caused by a deficient DNA mismatch repair (dMMR) system. Gastric cancers with MSI display an intrinsic high mutation load, providing neoantigens that increase sensitivity to immunotherapy (10). In a study encompassing 2545 patients including 123 with MSI gastric cancer, Pietrantonio et al. demonstrated that in MSI tumors, the hazard ratio for overall survival benefit from anti-PD-1 treatment was 0.34 (95% CI 0.21-0.54), whereas in microsatellite stable (MSS) tumors, the hazard ratio was 0.85 (95% CI 0.71-1.00), indicating the heightened responsiveness of MSI gastric cancer patients to immunotherapy (11). TMB is defined as the number of somatic nonsynonymous mutations within a specific genomic region, typically presented as the total number of mutations per megabase (mut/Mb). It has been observed to significantly correlate with objective response rate (ORR) and progression-free survival (PFS) in various cancers treated with immune checkpoint inhibitors (ICIs) (12–14).

While next-generation sequencing (NGS) and polymerase chain reaction (PCR) remain the gold standards for assessing microsatellite instability (MSI) and tumor mutational burden (TMB), their widespread clinical utility is significantly curtailed by high costs, prolonged turnaround times, and the requirement for substantial tissue samples. These limitations often preclude routine implementation in standard clinical workflows, particularly in resource-constrained settings. Consequently, it is imperative to develop accessible, cost-effective alternatives to optimize the management of gastric cancer (GC) patients.

Advances in computational histopathology have established that morphological patterns in pathological images can reflect underlying molecular alterations, positioning them as promising non-invasive proxies for predicting molecular and prognostic biomarkers in various cancers (15–19). Central to this approach are convolutional neural networks (CNNs), which enable large-scale processing and cross-referencing of histopathological data, facilitating the quantitative analysis of abnormal cellular and tissue structures (20, 21). Hematoxylin and eosin (H&E)-stained whole slide images (WSIs), in particular, capture phenotypic information at cellular and nuclear resolution, providing a rich data source for such analyses (22, 23). Several studies have already demonstrated the feasibility of using H&E-stained slides to predict biomarkers in multiple solid tumors (24, 25).

Deep learning (DL) algorithms have demonstrated transformative potential in medical image analysis. Emerging evidence suggests that histopathological phenotypes within standard hematoxylin and eosin (H&E)-stained whole slide images (WSIs) correlate closely with underlying genomic alterations. Consequently, the direct prediction of microsatellite instability (MSI) and tumor mutational burden (TMB) from WSIs has emerged as a rapid, cost-effective alternative to molecular sequencing. DL facilitates this process by autonomously extracting high-dimensional, discriminative features from expansive datasets, thereby uncovering subtle morphological patterns associated with specific disease states (26–28). Previous studies have successfully leveraged DL models to identify gene mutations directly from tumor tissues (29). Specifically, several research groups have applied these frameworks to predict MSI and TMB status (14, 17). For instance, Lee et al. developed a fully automated DL-based MSI classifier that achieved an area under the curve (AUC) of 0.892 using The Cancer Genome Atlas (TCGA) cohort (30). Similarly, in a multi-center retrospective study on gastric cancer (GC), Oliver et al. reported an AUC of 0.8092 for MSI prediction, validating the feasibility of ensemble learning approaches (31). Furthermore, Kather et al. established a robust model for MSI prediction in TCGA-STAD, achieving an AUC of 0.81 (95% CI, 0.69–0.90) (32). Despite the well-documented correlation between MSI and TMB, there remains a paucity of research focused on unified deep learning architectures capable of simultaneous dual-biomarker prediction—representing a critical gap in the current field of computational oncology.

In this study, we proposed a novel multi-task deep learning framework that integrates WSIs and clinical variables. We aim to establish an accessible pre-screening tool. Furthermore, we thoroughly evaluate the model on an expanded external independent cohort and provide quantitative interpretability analysis to ensure clinical reliability.

Initially, a pre-trained ResNet50 was utilized to extract features from the pathology images. Subsequently, feedback information from the MSI and TMB layers was incorporated using an attention mechanism, and the deep image features were fused with encoded clinical data to make the final prediction. Furthermore, the model visualizes crucial areas in WSI that are significant for MSI and TMB prediction through heatmaps. For additional insight from a pathologist’s viewpoint, cell nuclei image segmentation and classification were employed to further interpret the model’s functioning.

To evaluate the performance of our model and compare it with state-of-the-art models, we collected pathological image data and clinical information from the Cancer Genome Atlas (TCGA) and an independent validation cohort consisting of 121 GC patients enrolled at the Cancer Hospital of the Chinese Academy of Medical Sciences and Peking Union Medical College. Our aim was to present a potential alternative analytical approach for simultaneously determining MSI and TMB status, with a short diagnostic turnaround time and at a low cost.

## 2 Materials and methods

### 2.1 Study population and data collection

#### 2.1.1 Pathological image data

The data used in this study was composed of two distinct components: (1) TCGA: A total of 450 formalin-fixed and paraffin-embedded WSIs of GC patients were downloaded retrospectively from the TCGA public database for model training and internal validation, stained with hematoxylin and eosin. They were stored in “.svs” format. Additionally, the corresponding clinical data were obtained from TCGA and were loaded into the model as auxiliary information. By matching the H&E staining images with the clinical data, 312 cases with TMB tags were obtained, including 53 cases with TMB-H and 259 cases with low TMB (TMB-L). A total of 312 datasets containing MSI signatures were also obtained, including 55 MSI patients and 257 MSS patients. (2) Cancer Hospital, Chinese Academy of Medical Sciences: To address the requirement for robust external validation, we prospectively and retrospectively collected an expanded external cohort from the National Cancer Center. Initially, 130 patients were included (50 from our preliminary cohort and 80 newly enrolled patients). Following stringent quality control by a board-certified pathologist, 9 cases were excluded due to poor image quality (e.g., out of focus, folding) or lack of tumor tissue. The final external validation cohort comprised 121 patients. Clinical demographics (age, sex, TNM stage) and molecular ground truth (MSI status and TMB values via targeted NGS) were extracted from clinical records. Both tissue and blood samples underwent whole-exon sequencing to quantify somatic mutations and PCR methods for MSI detection. TMB was calculated using the Maftools R package (33), and the high TMB threshold was set at the 25th percentile for both TCGA and independent testing samples.

#### 2.1.2 Clinical data

We collected clinical data from public datasets and hospital-based clinical follow-up information. We cleaned the collected clinical data by removing clinical features that were not relevant to the research purpose, such as ID and version number. Additionally, we deleted clinical information with missing values greater than or equal to 25%, as severe missing can cause significant loss of data information, resulting in greater uncertainty and confusion in the data mining process and unreliable outputs. Subsequently, we matched the public and hospital-based clinical information and selected common clinical indicators as the input indicators for model training. We also filled the data according to the following rules: for discrete data, we used the median value for filling; for continuous data, we calculated the mean value of the data and filled in missing data based on these values. Ultimately, we selected six clinical indicators, namely gender, age, T stage, N stage, M stage, and number of lymph nodes resected. Additionally, while building the model, we conducted Cox regression analysis to estimate the relative risk of survival based on the selected clinical indicators and conducted rank sum tests to analyze differences based on MSI status and TMB status.

To demonstrate the comparability and representativeness of the data, the baseline clinical characteristics of the patients in both the TCGA training cohort and the independent hospital validation cohort are summarized in **Table 1**.

**Table 1:** Comparison results based on predictive MSI.

Model	AUC	Accuracy	Precision	Recall	F1-score
ResNet18	0.760	0.748	0.375	0.56	0.445
VGG16	0.696	0.672	0.324	0.643	0.421
VGG19	0.741	0.684	0.32	0.657	0.43
Ours	**0.828**	**0.807**	**0.444**	**0.68**	**0.557**

Bold values indicate the best performance among all methods.

### 2.2 Image preprocessing--DL framework

WSIs were digitized and fragmented into non-overlapping 256×256 pixel patches at 20× magnification. Background patches with<50% tissue area were excluded using Otsu thresholding. Color normalization was applied using the Macenko method to mitigate staining variations between TCGA and the external cohort.

**Figure 1:**
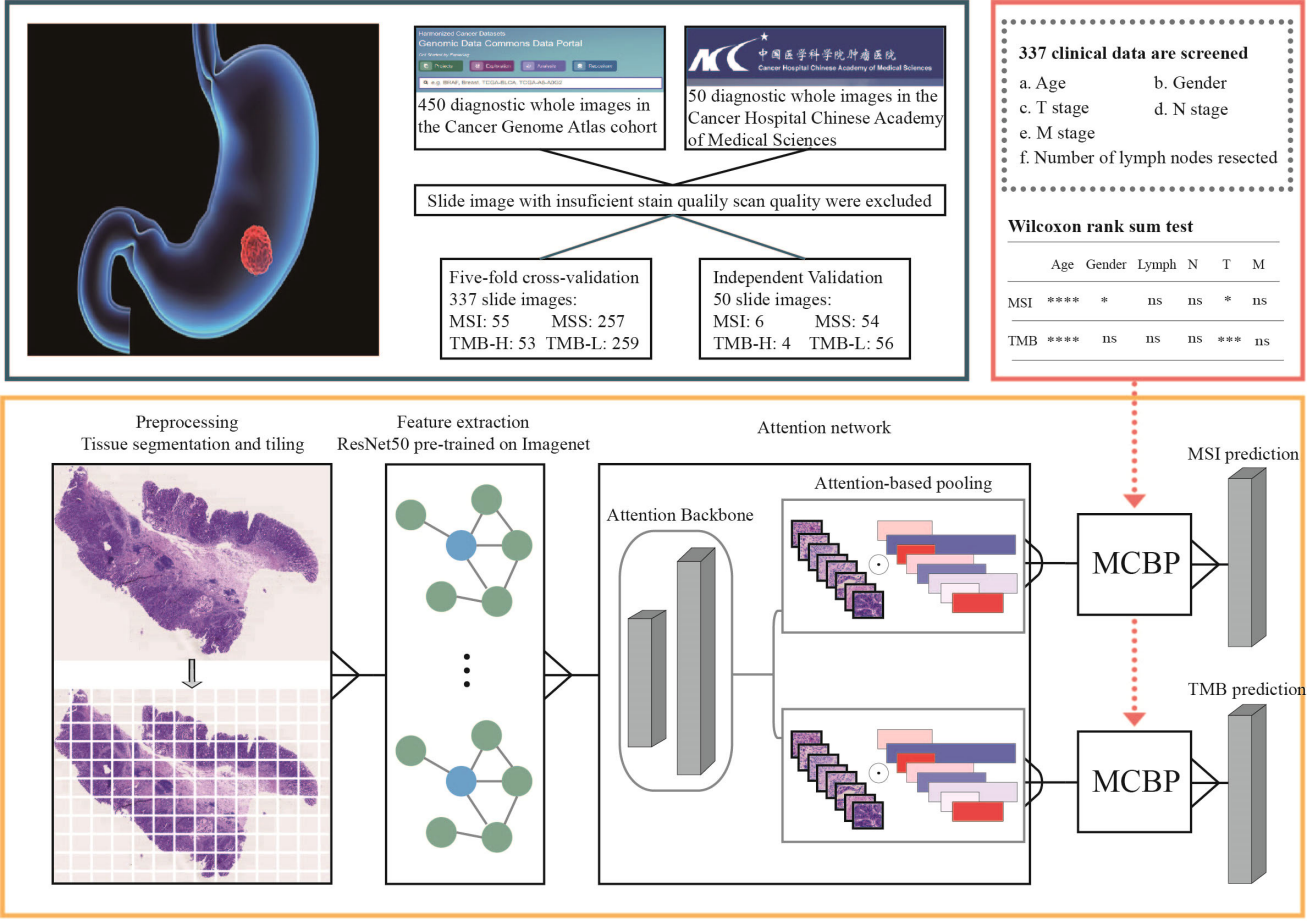
Flowchart.

We established a high-throughput DL-based network framework to develop a model that simultaneously predicted MSI and TMB in WSIs (**Figure 1**). In brief, firstly, based on the scanned H&E stained pathological images, a three-layer truncated ResNet50 residual network was used to automatically identify the tissue regions of the images and segment them into 256×256-sized image blocks. Each image block was encoded into a one-dimensional feature vector at the same time. The advantage of this approach was that it did not require manual marking of tumor regions, avoiding excessive reliance on subjective diagnosis by pathologists, while also reducing significant labor costs. Additionally, because WSIs are routinely used for clinical diagnosis, we used them to build predictive models. Subsequently, we trained the network framework through transfer learning and weak supervision learning, while utilizing attention modules to identify regions of rich information content in WSI. Finally, we linearly fused the deep image features and encoded clinical information through bilinear pooling, aggregating information before the final classification layer and obtaining the final prediction score. The model was trained using publicly available data from TCGA and optimized through five-fold cross-validation. Subsequently, the best model underwent external independent validation using hospital data.

### 2.3 Multi-task model architecture and multimodal fusion

A pre-trained ResNet50 was adopted as the backbone for feature extraction. We formulated a multi-task learning approach with shared bottom layers and two separate task-specific branches for MSI (classification) and TMB (regression/binary classification). To integrate clinical variables (age, sex, stage), we employed a Multimodal Compact Bilinear Pooling (MCBP) layer, capturing higher-order interactions between image features and tabular clinical data.

#### 2.3.1 Feature extraction

To extract effective information that could be provided to the model, the original pathology image was first subjected to semantic segmentation to extract the tissue regions of the image and remove background and other redundant information. The main operation involved down-sampling the entire pathology image in “.svs” format and converting it to HSV color space. Additionally, we smoothed edges, repaired holes, and thresholded the saturated channel image to form a binary mask of tissue regions. Finally, the approximate contour of the detected foreground object was filtered and stored based on the threshold region for downstream analysis. In the division, the extracted tissue region was cropped into 256×256 patches at a magnification of 20x, and stacks of image patches along with their coordinates and the slide metadata were stored using the hdf5 hierarchical data format.

We used a convolutional neural network (CNN) to calculate the feature representation of each pathology image. Specifically, we employed a truncated ResNet50 model pre-trained on ImageNet. To achieve this, the network was truncated after the third residual block (specifically, up to conv4_x layer), removing the subsequent conv5_x block and the fully connected classification layers. This modification allowed us to directly encode each 256 x 256 x 3 patch into a 1024-dimensional feature vector. Furthermore, to implement transfer learning effectively, the pre-trained weights were strictly frozen during this feature extraction process to preserve the generalized visual representations learned from ImageNet, thereby preventing overfitting on our specific dataset. Subsequent training was conducted in a fixed low-dimensional feature space rather than in pixel space, significantly reducing time costs and computational resources.

#### 2.3.2 Attention module-based training mechanism

The training mechanism based on attention modules mainly involved processing the input data through a series of linear and nonlinear layers, attention mechanisms, followed by forward propagation, calculation of output and error, backward propagation, and gradient update. The attention module is a method used in DL models to improve model performance. It focuses on key regions or features in the input data to enhance the model’s feature extraction and representation capabilities for these regions, which helps improve the model’s performance and generalization ability. To better train the network, enable the model to automatically locate regions of high diagnostic relevance in the slide, and aggregate their information to make final predictions, we extended the attention-based information pooling to multiple tasks, aggregated information from all tissue regions in each WSI, and outputted final slide-level predictions on this basis. The specific procedures were as follows:

1. By utilizing two fully connected layers to transform the learned deep image features into histologically specific feature representations, we map the given WSI image block feature set into a 512-dimensional feature vector.

Multi-task attention pooling: in the multi-task attention framework used, the multi-layer attention module consisted of two layers, Attn-Fc1 and Attn-Fc2, with weight parameters of $V_{a}\in R^{384\times 512}$ and $U_{a}\in R^{384\times 512}$ for each layer, while the weight parameter was shared between the two tasks. Each task had a separate set of weights $W_{a,t}\in R^{1\times 314}$, used for specific task training, and then the attention score *a_k,t_* activated by Softmax was assigned to each patch of WSI.

2. The attention pool represents the deep histological features of each task’s entire WSI by calculating the weighted average of the feature representations of all patches in the slide, based on their respective predicted attention scores *a_k,t_*.

#### 2.3.3 Multimodal Compact Bilinear Pooling

MCBP obtains a compact cross-modal feature representation by bilinear pooling input feature maps from different modalities. Specifically, MCBP obtains a single pooled feature vector through weighted fusion of feature maps from different modalities and bilinear pooling of the fused feature maps. The length of this pooled feature vector is usually relatively short, indicating the importance of the input feature map in cross-modal space, effectively improving the model’s performance and generalization ability. Additionally, by using linear kernel machines to encode clinical data and weighted fusion of deep image features at the final classification layer of each task, the data was finally input into the Softmax function to achieve classification prediction.

#### 2.3.4 Hyperparameters and model selection

We used a five-fold cross-validation method to train and tune the model. During training, the slides were randomly sampled and provided to the model in a batch size of 1, and a multi-task objective was used to supervise the neural network during training. The weights and bias parameters of the attention module were randomly initialized and trained end-to-end with the rest of the model using slide-level labels. For each WSI, the total loss was the weighted sum of the losses incurred by the first task of predicting MSI and the second task of predicting TMB, as shown in **Equation 1**:

(1) $${ℒ}_{total}=c_{1}{ℒ}_{MSI}+c_{2}{ℒ}_{TMB}$$

The standard cross-entropy loss function was used for both tasks, and c1 = c2 = 0.5 was applied. The model parameters were updated using the stochastic gradient descent (SGD) optimizer with a learning rate of 2 × 10^−3^ and a weight decay of 1 × 10^−5^. To prevent potential overfitting of the model, we also employed a dropout layer with P = 0.25 after each hidden layer. All models were trained for at least 100 epochs, and if the early stopping criteria were not met, they were trained for up to 300 epochs. In other words, each epoch monitored the validation loss, and when the validation loss did not decrease from the previous low point for more than 121 consecutive epochs, early stopping was used. The saved model had the lowest validation loss, and then it was tested on the test set.

#### 2.3.5 Ablation studies and baseline comparison

To rigorously evaluate our architectural choices, we designed comprehensive baseline comparisons and ablation studies. We compared our ResNet50 backbone against standard architectures including ResNet18 (34) and VGG series such as VGG16 and VGG19 (35). ResNet18 is a CNN model that is primarily characterized by the introduction of the concept of the residual block. This design was intended to address the issues of gradient vanishing and gradient explosion encountered in deep CNNs. Furthermore, ablation studies were conducted to assess the individual contributions of: (1) Single-task vs. Multi-task learning; and (2) Image-only vs. Multimodal (Image + Clinical data) approaches.

In the residual block, the shortcut connection allows direct connection between the input to the output, enabling the network to learn residual information and perform better feature extraction and processing. Additionally, ResNet18 adopts various optimization techniques such as SGD and Momentum optimization algorithm, which can enhance accuracy and robustness. The VGG series is a CNN model whose design philosophy is to construct deep networks by stacking multiple smaller convolutional layers and pooling layers to enhance the model’s expressive power. The VGG16 model was trained using a smaller learning rate with momentum gradient descent to optimize the objective function.

### 2.4 Model visualization and cell identification

#### 2.4.1 Attention visualization

We used attention heat maps to visualize the model prediction process in order to better understand the model’s focus and decision-making process when processing input data, visually explaining the importance of each region in the WSI for model classification and prediction. First, the attention score obtained for each patch was used as a reference, and the repeated tiling of the single patch was maximally overlapped by 95%. The attention score after the overlap was converted to a normalized percentage between 0 and 1, and the normalized score was recorded at the original WSI position corresponding to each patch. Finally, the color map was mapped to the attention score.

### 2.5 Interpretability and quantitative spatial analysis

#### 2.5.1 Interpretability analysis

**Figure 2:**
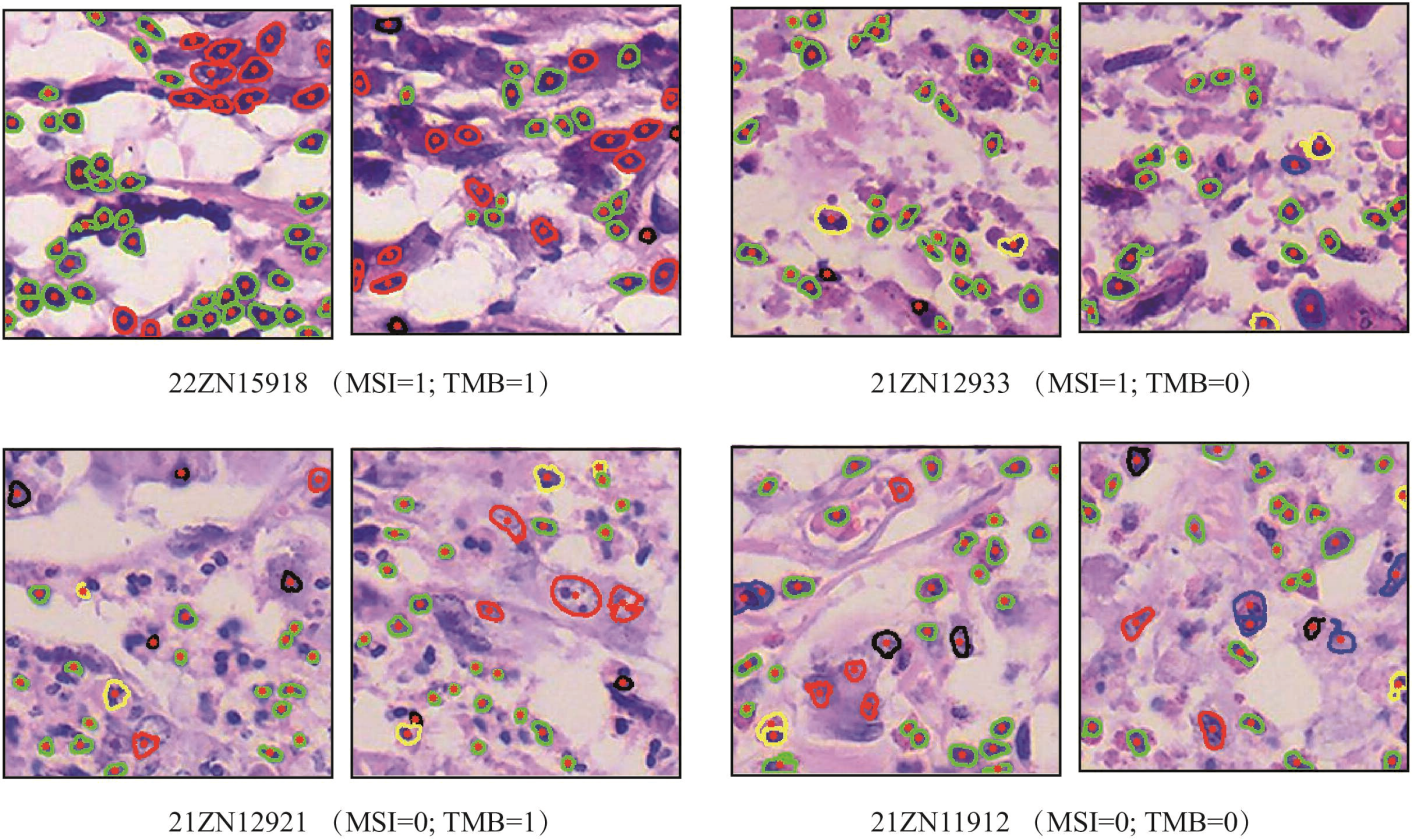
Cells segmented using the hover-net model. In the figure, black represents epithelial cells; red represents tumor cells; green represents lymphocytes; blue represents stromal cells; orange represents necrotic cells.

Interpretability was achieved using attention mechanisms to generate heatmaps. To overcome superficial visual evaluation, we introduced a quantitative spatial analysis. We calculated the Intersection over Union (IoU) and Pearson spatial correlation coefficient between the top 10% highest-attention regions for MSI and TMB tasks. Furthermore, a senior pathologist blindly evaluated the top 1% high-attention patches to identify corresponding histological features (e.g., tumor-infiltrating lymphocytes (TILs), mucin pools, necrosis) using the Hover-Net segmentation framework (**Figure 2**).

#### 2.5.2 Quantitative analysis of high-concentration regions

We conducted quantitative analysis of cell populations within high-concentration regions of all MSI states and TMB states in the test set using a quantitative model. Specifically, the top 10 high-attention plaques on each slide were extracted at a magnification of 20x, and the HoverNet model was trained for multi-type cell nucleus segmentation. Classification was used to identify different cell populations, including tumor cells, lymphocytes, connective tissue, dead cells, and non-tumor epithelial cells.

#### 2.5.3 Evaluations metrics

In this investigation, the metrics used to assess the relative merits of the prediction model were: AUC, accuracy, precision, recall and F1-score. AUC was defined as the area under the ROC curve bounded by the x-axis, and this value ranged from 0 to 1. The computational formula of accuracy is listed in **Equation 2**, precision is defined in **Equation 3**, recall is shown in **Equation 4**, and the F1-score is calculated via **Equation 5**:

(2) $$\text{Accuracy}=\frac{TP+TN}{TP+TN+FP+FN}$$

(3) $$\text{Precision}=\frac{TP}{TP+FP}$$

(4) $$\text{Recall}=\frac{TP}{TP+FN}$$

(5) $$\text{F}1\text{-score}=2\frac{precision\times recall}{precision+recall}$$

where TP is the number of samples correctly predicted as positive, TN is the number of samples correctly predicted as negative, FP is the number of samples incorrectly predicted as positive, and FN is the number of samples incorrectly predicted as negative.

## 3 Results

### 3.1 Patient characteristics

In the TCGA cohort (n=312), the median age was 67 years. In the expanded external independent cohort (n=121), the median age was 63 years (range 42-81); 82 (67.8%) were male and 39 (32.2%) were female. Regarding molecular status, 24 patients (19.8%) were identified as MSI-H, and the median TMB was 6.5 mutations/Mb.

### 3.2 Baseline model comparison and ablation study

**Figure 3:**
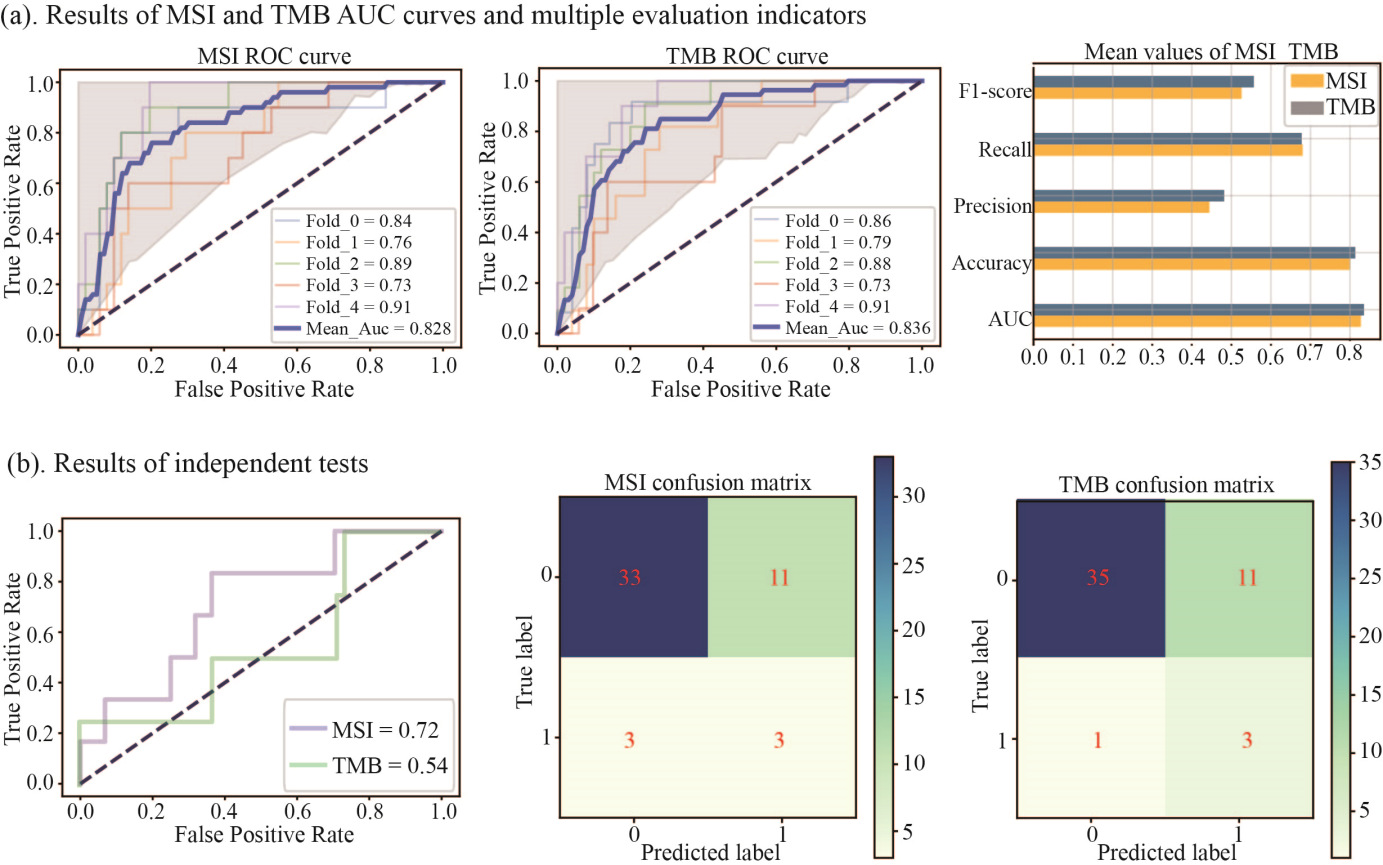
Performance evaluation results of models built based on images only during training and external independent testing. **(a)** AUC values and other evaluation metrics for model 5-fold cross-validation on public datasets. **(b)** Evaluation results on external independent test data.

**Figure 4:**
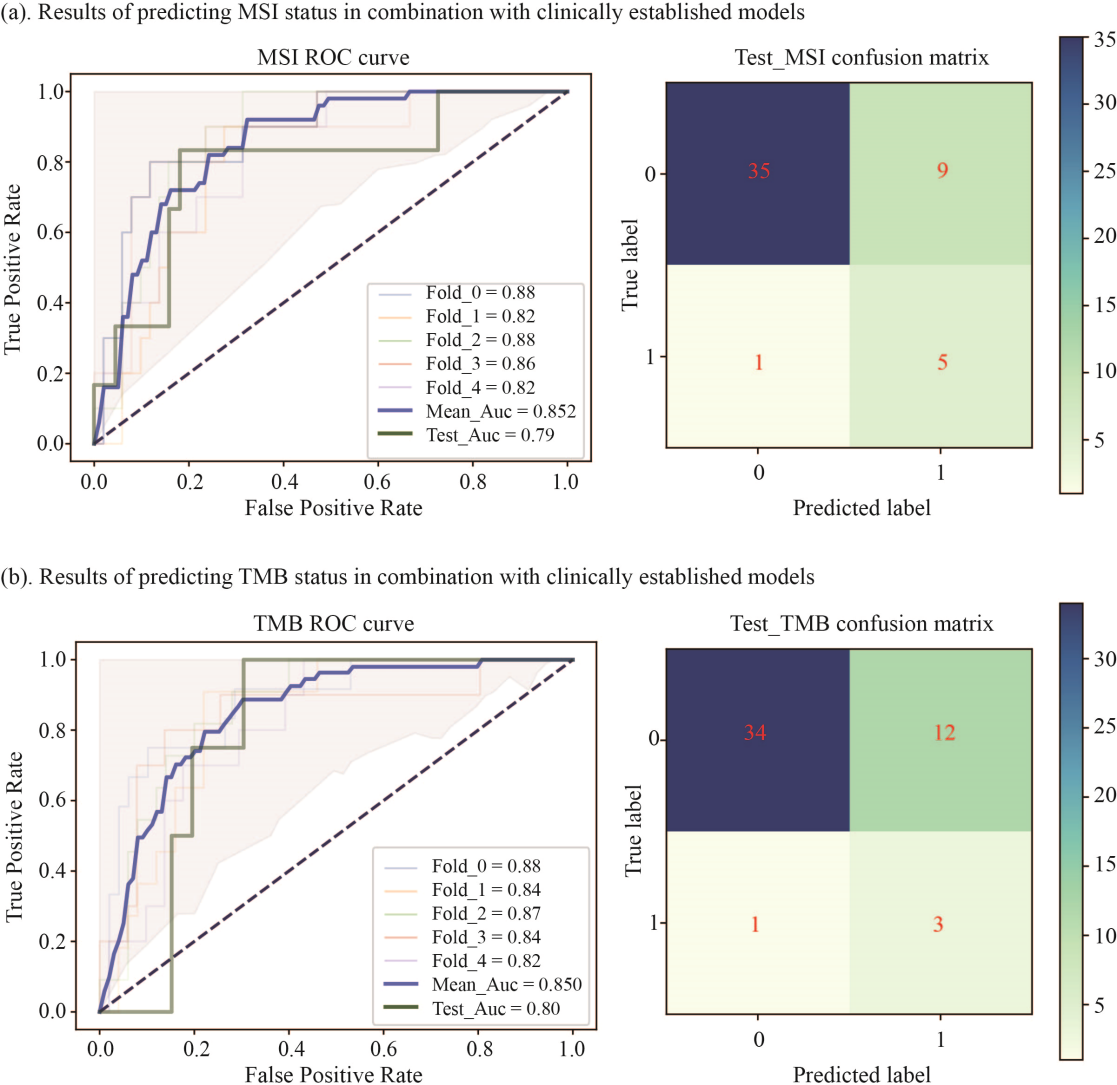
Test results of fusing image features with clinical information on TCGA and independent datasets. **(a)** ROC curve based on MSI and confusion matrix on independent testing data; **(b)** ROC curve based on TMB and confusion matrix on independent testing data.

We compared the proposed ResNet50-based model against VGG16, VGG19, and ResNet18. ResNet50 outperformed VGG16 (AUC 0.72) and ResNet18 (AUC 0.79) with a baseline AUC of 0.83 for MSI prediction on the internal validation set, justifying its selection as the backbone. Ablation studies confirmed the superiority of our framework. The multi-task model (simultaneous MSI/TMB) demonstrated a 4.5% improvement in overall accuracy compared to single-task models, indicating that shared feature representations benefit both related biomarkers. Furthermore, integrating clinical data via MCBP yielded a statistically significant increase in AUC (p<0.05) compared to the image-only model (**Figures 3a**, **4**).

### 3.3 Predictive performance of models based on histopathology images from internal and external validation

The multimodal framework achieved high predictive performance during 5-fold cross-validation on the TCGA dataset. We leveraged data collected from 312 patients in TCGA, which included both MSI and TMB labels and corresponding histopathological images. Among them, there were 53 MSI cases and 55 TMB-H cases. We used these public datasets for model training and parameter tuning.

**Figure 5:**
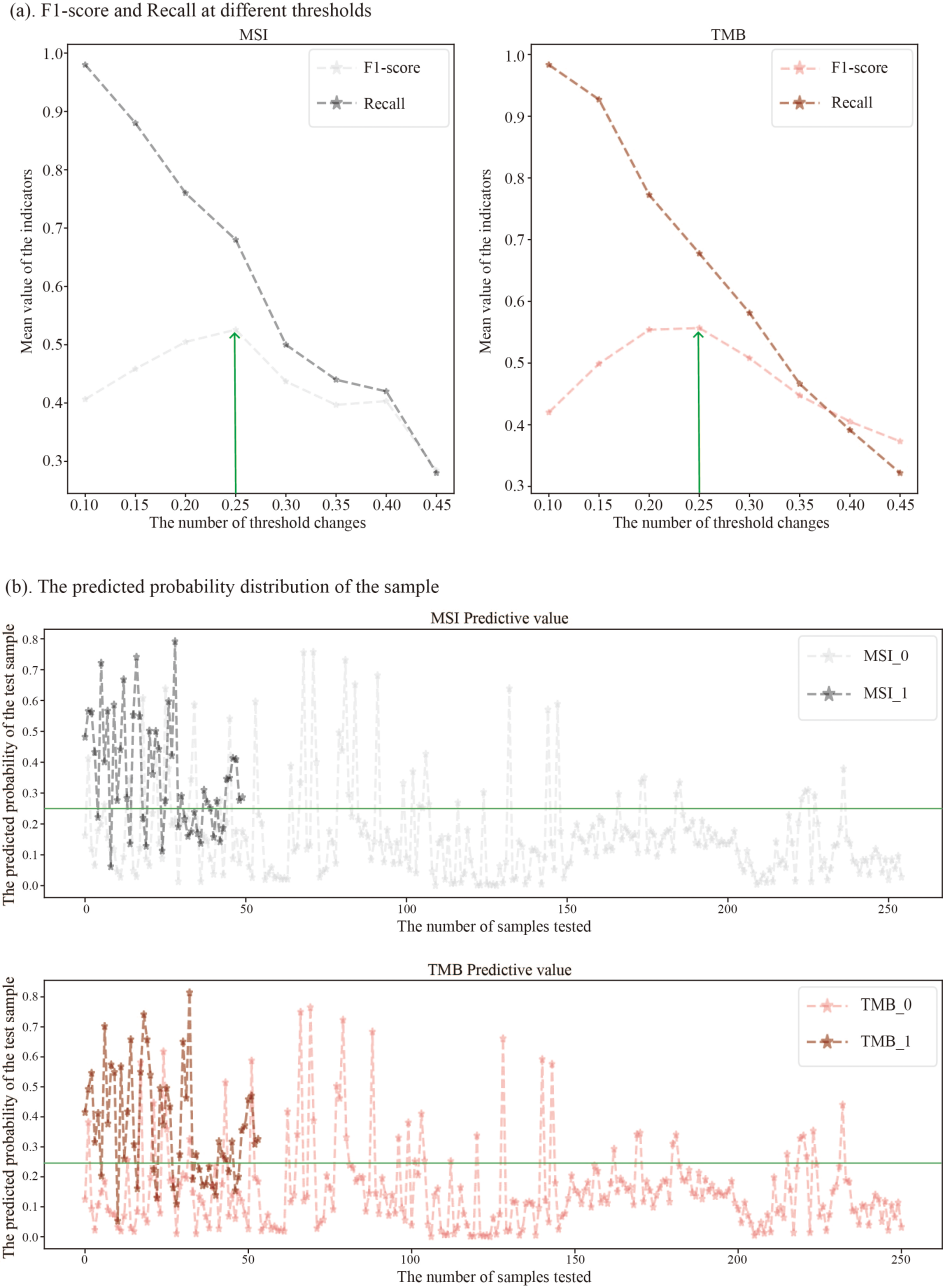
Probability line graph based on different thresholds. **(a)** The line graphs of the F1-score and Recall values of MSI and TMB calculated based on a step size of 0.05, with the green arrow indicating the optimal threshold selected. **(b)** The distribution diagram of the predicted values of test samples based on MSI and TMB, where MSI_ 0 indicates MSS, MSI_ 1 represents MSI, TMB_ 0 represents TMB_ L, TMB_ 1 represents TMB_ H.

In the field of medical image analysis, if we simply used accuracy as the final evaluation metric, we might obtain misleading information in some cases. This is because in scenarios where the sample data is imbalanced, the model’s predictions tend to be biased toward the side with a higher sample ratio. However, the proportion of MSI in GC is around 5.6%, which is insufficient to accurately detect MSI status in a timely manner. Therefore, we introduced metrics such as precision, recall, and F1-score, and selected the optimal threshold to assist the model in reporting classification performance. We gradually selected the appropriate threshold value by taking steps of 0.05, as shown in **Figure 5a**. When the threshold value was 0.25, the F1-score for predicting TMB and MSI calculations were the highest. The reason why we use F1-score as a limiting indicator is that precision and recall are contradictory measures. When the classification confidence is high, precision is high; when the classification confidence is low, the recall is high. In order to comprehensively consider these two indicators, the F1-score is introduced. Its core idea is to maximize both precision and recall while minimizing the difference between them. In addition, the prediction probability line graph of MSI (TMB-H)/MSS (TMB-L) displayed in **Figure 5b** shows that when the threshold is set to 0.25, several states can be distinguished to the greatest extent.

### 3.4 Quantitative interpretability and histological validation

The prediction results based on the network framework using five-fold cross-validation training were used for parameter tuning and performance evaluation of the model. The prediction results on the public data are shown in **Figure 3a**. The results showed that the AUC score of the model for predicting MSI status reached 0.828, and the AUC for predicting TMB status reached 0.836.

In addition, other metrics were calculated under a threshold value of 0.25, including sensitivity and positive predictive value (PPV) of MSI status prediction, which were 0.68 and 0.80, respectively, and sensitivity and PPV of TMB status prediction, which were 0.677 and 0.813, respectively.

However, when applied to the external independent test data (n=121), the model experienced a performance drop, yielding an AUC of 0.78 for MSI and 0.74 for TMB. This decrease reflects the inherent challenges of domain shift, including variations in tissue preparation, scanner hardware, and cohort demographics between the public TCGA data and our local center (**Figure 3b**).

**Figure 6:**
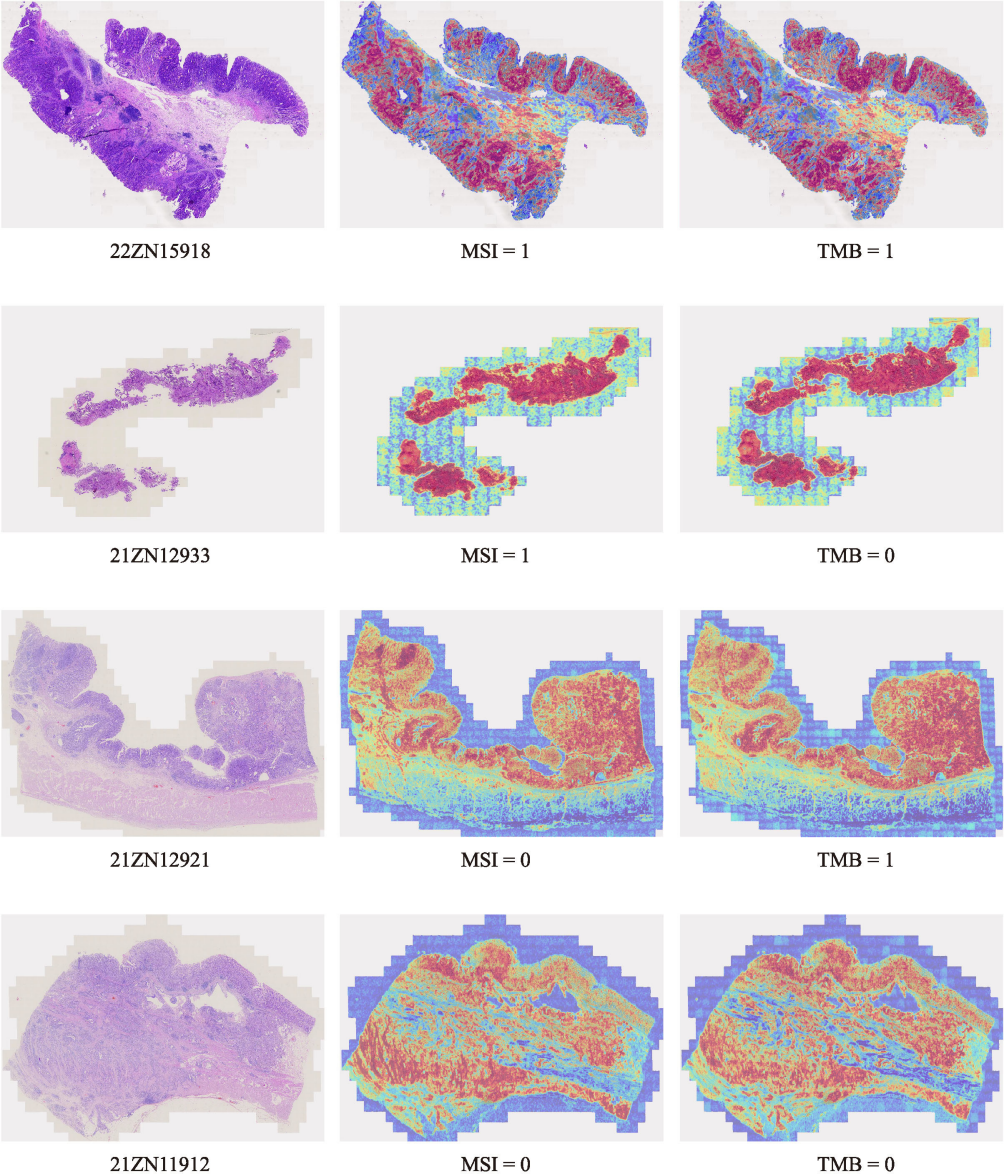
Heatmap based on model-predicted MSI and TMB. MSI = 0 represents MSS, MSI = 1 represents MSI; TMB = 0 represents TMB-L, TMB = 1 represents TMB-H.

We visualized the attention process of each WSI in the model for human interpretation and validation. We grouped the samples according to different MSI and TMB status, and plotted MSI heatmaps and TMB heatmaps for the same patients in the test set. As shown in **Figure 6**, attention heatmaps localized the predictive regions. Quantitative analysis of these maps revealed a high spatial concordance between MSI and TMB predictive regions, with a mean Pearson correlation coefficient of 0.72 and an IoU of 0.65 for the top 10% attention areas.

Crucially, histological validation by a board-certified pathologist using Hover-Net cell segmentation (**Figure 2**) confirmed that these high-attention regions were not random. Regions predictive of MSI-H and high TMB were significantly enriched with tumor-infiltrating lymphocytes (TILs, green cells) and necrotic areas (orange cells), which aligns with established biological knowledge of highly immunogenic tumors.

We compared our method with several models commonly used for image classification training, including ResNet18, VGG16, and VGG19. Due to the severe imbalance in the positive and negative sample ratio of the external independent data and the small number of positive samples, we conducted the entire comparative experiment only on public data. During the experiment, in order to ensure fairness, we used the same split datasets and hyperparameters. We also conducted five-fold cross-validation for parameter tuning during the training process of the comparative models. Finally, the results were evaluated using the metrics described in Section 2.3.

**Tables 1**, **2** show the prediction results of MSI and TMB, respectively, in this comparative experiment. The results revealed that the model we employed excelled in TMB prediction and MSI prediction, surpassing models such as ResNet18, VGG16, and VGG19. Compared to these models, our model exhibited higher accuracy, lower error rates, and superior robustness. Additionally, a joint examination of **Tables 1**, **2** indicated that the model possessed comparable accuracy in predicting TMB and MSI, further corroborating the close correlation between TMB and MSI in gastric cancer. This finding may pave new paths for immune therapy in gastric cancer patients in the future.

**Table 2:** Comparison results based on predictive TMB.

Model	AUC	Accuracy	Precision	Recall	F1-score
ResNet18	0.749	0.778	0.441	0.599	0.497
VGG16	0.766	0.688	0.333	0.600	0.428
VGG19	0.736	0.684	0.358	0.660	0.460
Ours	**0.836**	**0.813**	**0.482**	**0.678**	**0.557**

Bold values indicate the best performance among all methods.

**Figure 7:**
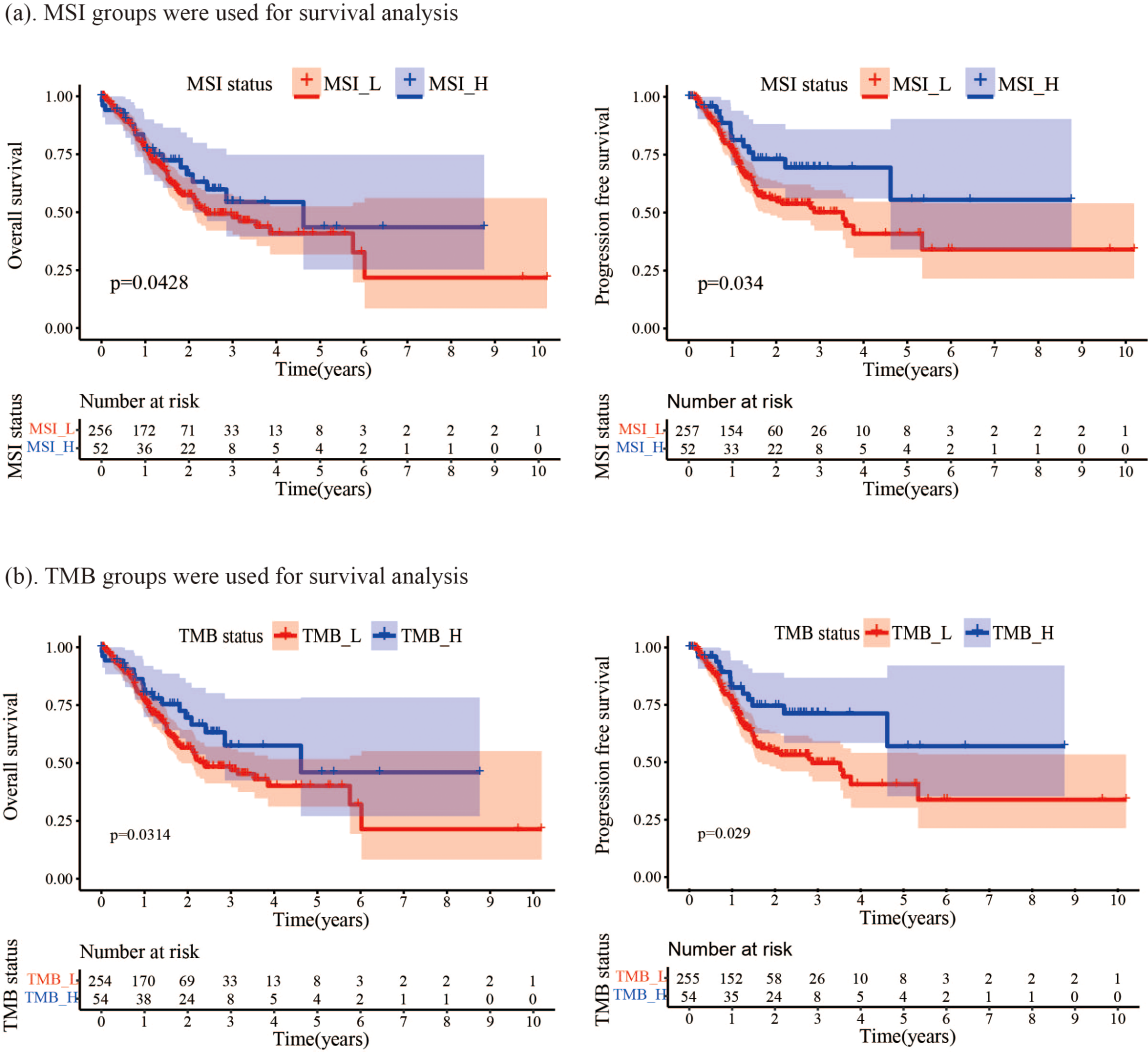
K-M curve on MSI and TMB groups. **(a)** Survival curve and relapse-free survival curve on MSI. **(b)** Survival curve and relapse-free survival curve on TMB.

In addition, to explore the impact of MSI and TMB on patient prognosis, we matched the corresponding clinical survival indicators based on the predicted MSI and TMB status, and analyzed the patient’s survival rate and relapse-free survival rate using the K-M curve. As shown in **Figure 7**, there were significant differences in patients’ conditions.

### 3.5 Prediction performance of multimodal model that fuses image information and clinical information

**Figure 8:**
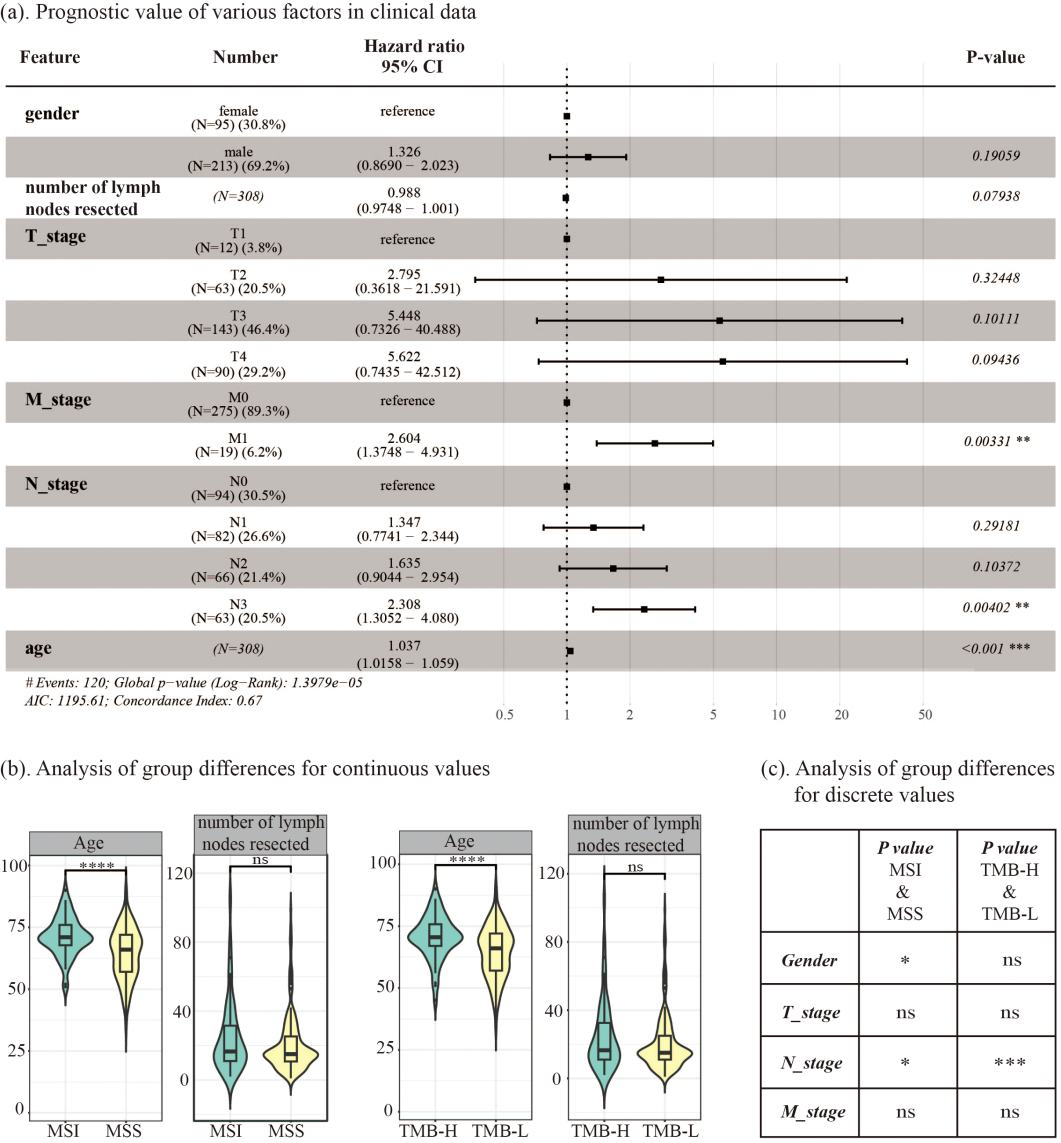
Clinical statistical analysis. **(a)** Single-factor COX regression was used to assess the risk of clinical indicators on survival outcome; **(b)** Wilcoxon rank-sum test for continuous values; **(c)** Wilcoxon rank-sum test for discrete values.

We matched the clinical data with the pathological images of TCGA, and finally selected six clinical characteristics: T stage, N stage, M stage, age, number of lymph nodes resected, and gender, by screening the data that overlapped with the hospital. We used univariate COX regression to analyze six clinical characteristics and evaluate the potential consistency of clinical indicators. As shown in **Figure 8a**, in all subgroups analyzed, age (P<0.001), M1 stage (P<0.005), and N3 stage (P<0.005) were significantly associated with the survival outcome of GC patients. In addition, we conducted statistical analysis on the clinical characteristics of different MSI status and TMB status (**Figures 8b, c**). We used the Wilcoxon rank-sum test to analyze the differences in the characteristics of the groups. The analysis results showed that age and N stage had significant effects on both MSI status and TMB status.

We then used the MCBP algorithm to fuse clinical and image features during model training in order to improve the prediction performance of the model through the integration of multi-dimensional information. The results are shown in **Figure 4**: the average results of the multimodal combination model of image and clinical data had significantly improved on the five-fold cross-validation (MSI: 0.828/0.852, TMB: 0.836/0.850). In addition, in the confusion matrix of independent testing, the MSI status and TMB-H prediction accuracy had also been further improved. It indicates that multimodal information fusion can indeed fill the information gap of a single mode and improve the generalization ability and prediction accuracy of the model at a lower cost.

## 4 Discussion

In this study, we successfully developed and validated a multimodal, multi-task deep learning framework designed for the simultaneous prediction of microsatellite instability (MSI) and tumor mutational burden (TMB) directly from routine H&E-stained whole slide images (WSIs) and clinical data in gastric cancer (GC). The predictive value of TMB and MSI in response to immune checkpoint inhibitors (ICIs) as adjuvant therapy is widely recognized. However, identifying these biomarkers remains a significant clinical challenge. Traditional approaches, such as next-generation sequencing (NGS) and polymerase chain reaction (PCR), often incur high costs, demand substantial tissue samples, and cause treatment delays. Overcoming the limitations of current computational methods that primarily focus on isolated marker status, our integrated screening tool addresses the critical need for efficient evaluation of interconnected genomic instability and immune responses. By leveraging multi-task learning, our model exploits the underlying biological correlation between mismatch repair deficiency and subsequent mutational accumulation, demonstrating enhanced predictive capability over single-task models like ResNet18 and VGG16. Furthermore, quantitative spatial attention analysis (IoU = 0.65) confirmed that the network utilizes shared morphological features, successfully colocalizing with established immunogenic hallmarks such as dense tumor-infiltrating lymphocytes (TILs), necrosis, and mucinous differentiation, thereby providing necessary biological plausibility to the model.

Despite robust internal validation, we acknowledge a substantial reduction in predictive accuracy when applying the model to our external validation cohort, particularly for TMB prediction (AUC drop from >0.85 to ~0.78). Rather than claiming absolute generalizability, we recognize this attenuation as a common yet critical bottleneck in computational pathology. This performance discrepancy is highly sensitive to pre-analytical domain shifts, including variations in tissue fixation, H&E staining protocols, and digital scanner profiles between source centers and the external hospital. Additionally, this drop reflects inherent biological and ethnic heterogeneity between the predominantly Western TCGA training dataset and our Asian external validation cohort. The external evaluation was also severely underpowered by a small sample size with a restricted number of MSI and TMB-H positive cases, restricting statistical confidence and precluding definitive conclusions. Furthermore, due to the retrospective nature of the data, the availability of comprehensive clinical variables was limited.

While our study demonstrates the theoretical feasibility and morphological basis of simultaneously predicting multiple genomic biomarkers, it highlights profound challenges regarding model generalizability across different clinical centers. To prevent overfitting to source-specific imaging artifacts, future research must incorporate advanced domain adaptation strategies, such as generative adversarial network (GAN)-based stain transfer or federated learning. A realistic pathway to clinical implementation will necessitate training and validating the framework on large-scale, multi-center cohorts with balanced positive cases and robust statistical significance testing. Ultimately, from a clinical perspective, we do not propose this model as a complete replacement for NGS. Instead, it holds significant potential as a rapid, cost-effective pre-screening triage tool. In resource-limited settings, rapidly screening all GC resections could efficiently prioritize high-probability MSI-H/TMB-H patients for confirmatory NGS and subsequent immunotherapy, thereby optimizing medical resources and reducing turnaround times.

## 5 Conclusion

In conclusion, we have developed a multimodal deep learning framework capable of simultaneously predicting MSI and TMB in gastric cancer using histopathological images and clinical data. While our approach shows promise in highlighting the correlation between tumor morphology and genetic biomarkers, the current model exhibits performance variability across different institutional datasets. Further validation on larger, multi-center cohorts and the integration of advanced domain adaptation strategies will be required before this framework can be reliably integrated into routine clinical decision-making workflows.

## Data Availability

The datasets used and/or analyzed during the current study are available at https://github.com/lvyaping/yiz_gastric_TMB_MSI.
